# Supplementary material for: Independently evolved viral effectors convergently suppress DELLA protein SLR1-mediated broad-spectrum antiviral immunity in rice
Source: Nat Commun. 2022 Nov 14;13:6920. doi: 10.1038/s41467-022-34649-z (PMC9663503; doi:10.1038/s41467-022-34649-z)
Supplement: Supplementary file 1 — Supplementary Information [file 41467_2022_34649_MOESM1_ESM.docx]

Supplementary Information for

**Independently evolved viral effectors convergently suppress DELLA protein SLR1-mediated broad-spectrum antiviral immunity in rice**

Lulu Li^1,2^, Hehong Zhang^2^, Zihang Yang^2^, Chen Wang^1,2^, Shanshan Li^1,2^, Chen Cao^2^, Tongsong Yao^1,2^, Zhongyan Wei^2^, Yanjun Li^2^, Jianping Chen^1,2^* and Zongtao Sun^2^*

^1^College of Plant Protection, Nanjing Agricultural University, Nanjing 210095, China.

^2^State Key Laboratory for Managing Biotic and Chemical Threats to the Quality and Safety of Agro-products, Key Laboratory of Biotechnology in Plant Protection of MOA of China and Zhejiang Province, Institute of Plant Virology, Ningbo University, Ningbo, 315211, China.

*Authors for correspondence:

Jianping Chen, jianpingchen@nbu.edu.cn;

Zongtao Sun, sunzongtao@nbu.edu.cn

**Supplementary Figures: 15**

**Supplementary Table: 1**

**Supplementary Figures 1- 15**

**
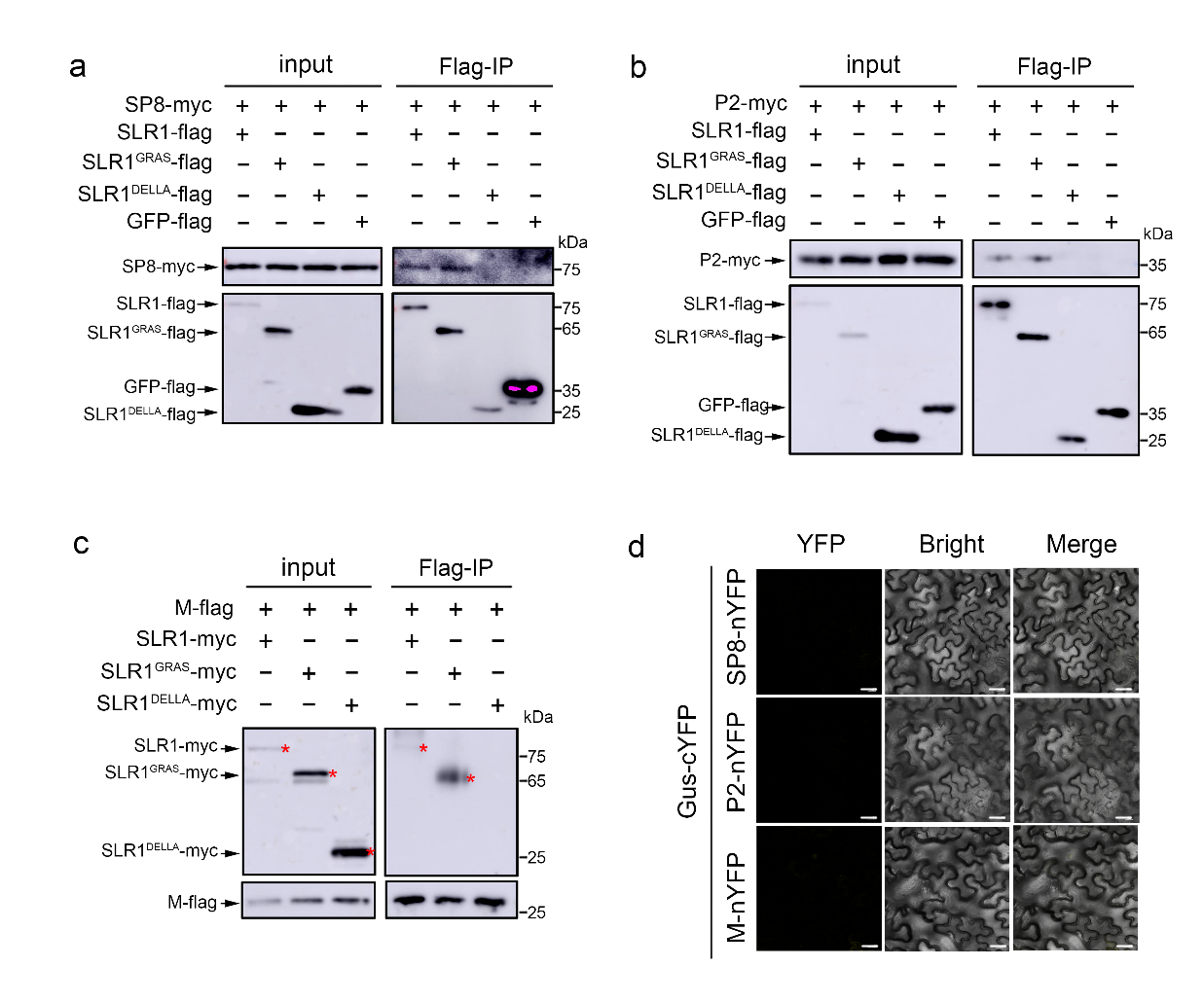
**

**Supplementary Figure 1. Interaction among SLR1 with SP8, P2 and M protein. (a-c)** Co-IP assay mapped the domain of SLR1 interacting with SP8 **(a)**, RSV P2 **(b)**, and RSMV M protein **(c)**. **(d)** BiFC assays examined that viral proteins SP8, P2 and M could not restore the YFP fluorescence with Gus-cYFP. Viral proteins SP8, P2 and M, fused with cYFP vector, agro-injected together with SLR1-nYFP or Gus-nYFP alone into *N. benthamiana* leaves, where co-infiltrated leaves were treated without MG132, and then imaged by confocal microscopy at 48 hpi. Scale bar = 20 µm.

**
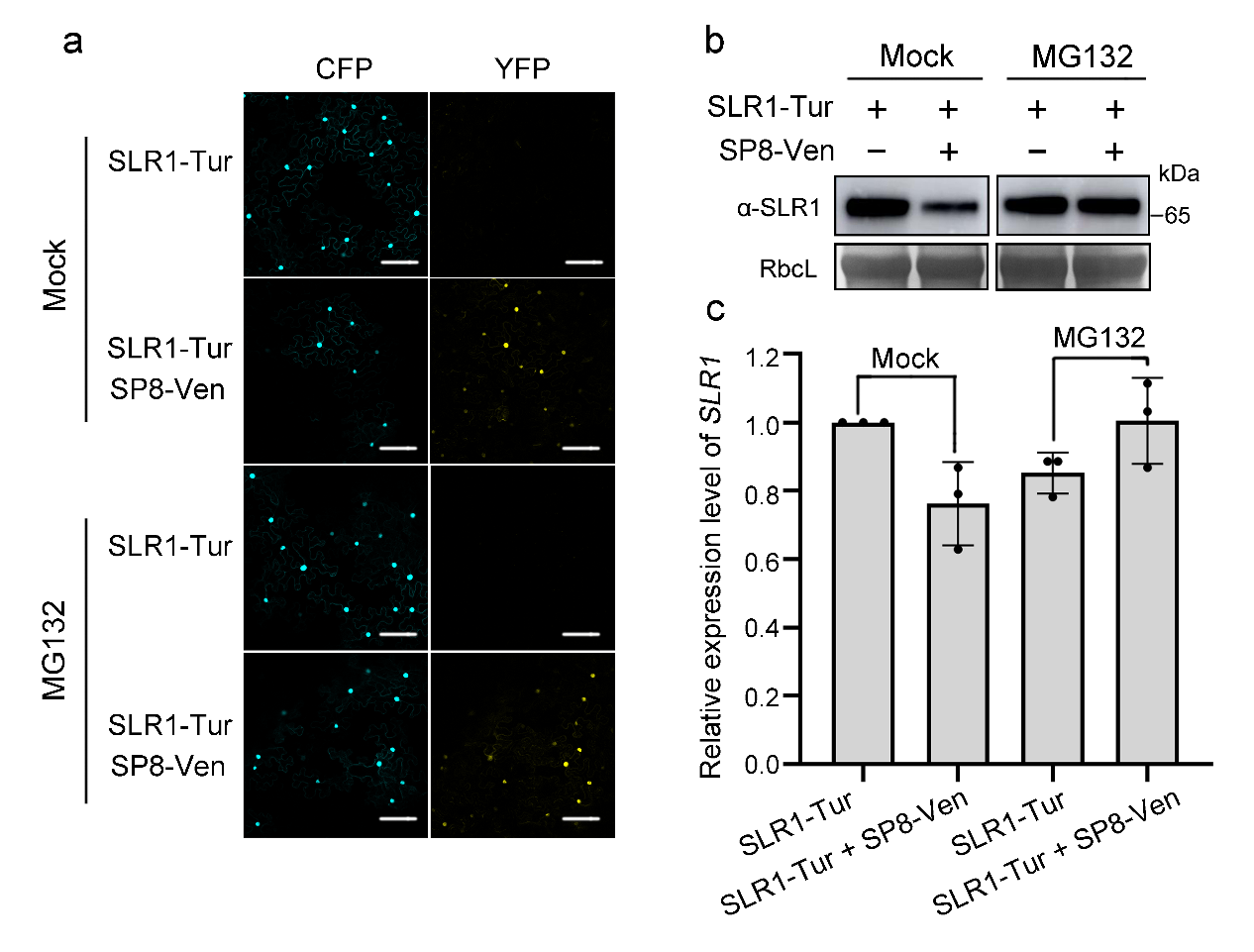
**

**Supplementary Figure 2. SP8 and RSV P2 provoke rapid degradation of SLR1.** **(a)** BiFC assays confirming that viral protein SP8 affects the accumulation of SLR1 in *N. benthamiana* leaves. SLR1-Tur was agro-injected together with SP8-Ven or alone into *N. benthamiana* leaves, where co-infiltrated leaves were treated with MG132 (50 µM) or DMSO at 24 hpi and then imaged by confocal microscopy at 48 hpi. Scale bar = 50 µm. **(b)** Western blot showing the levels of SLR1 protein in the leaves from (a). RbcL was used as a loading control. **(c)** Results of qRT-PCR showing the SLR1 mRNA levels in the leaves from (a).

**
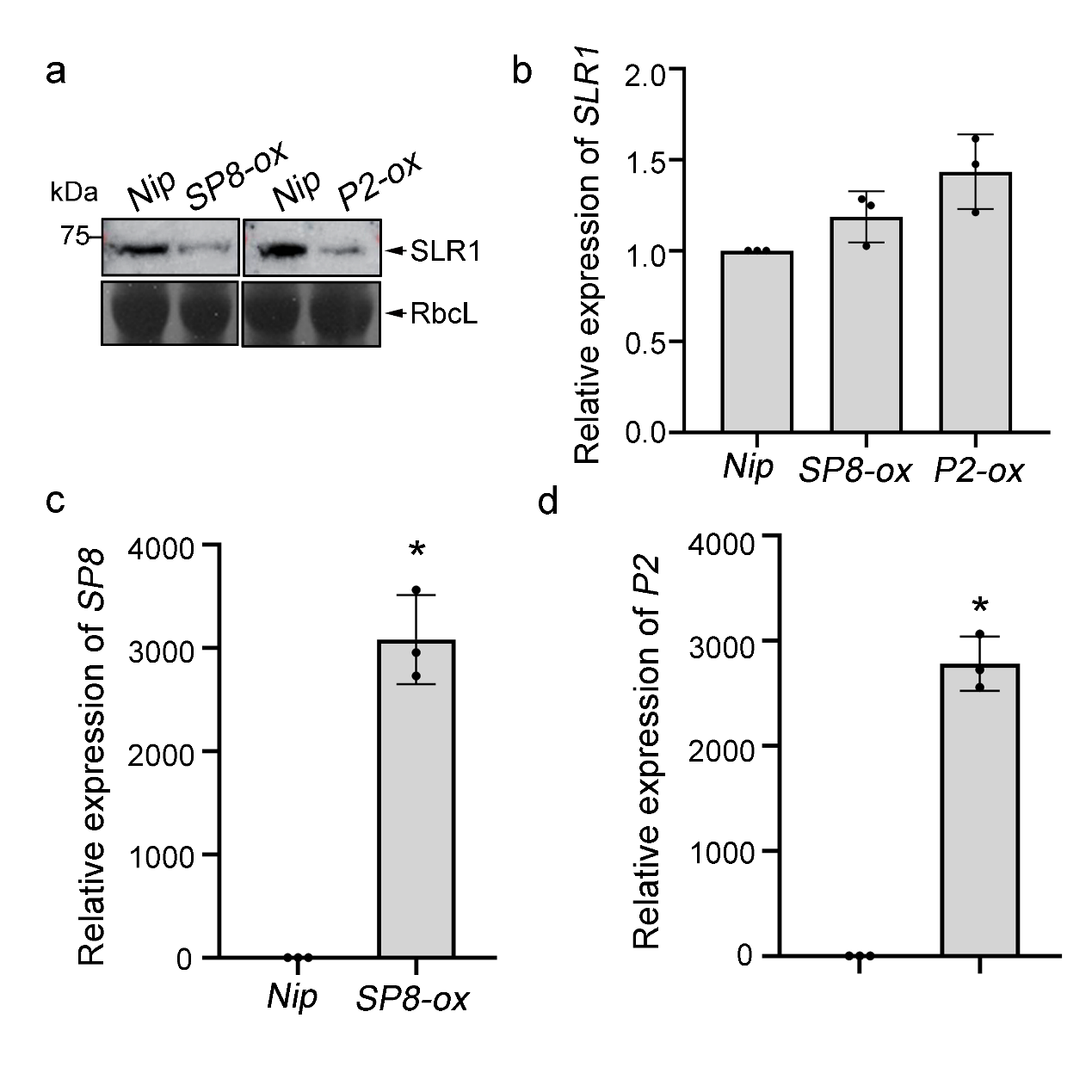
**

**Supplementary Figure 3. Viral protein promote degradation of endogenous SLR1 in transgenic rice plant. (a)** Endogenous SLR1 protein levels in *SP8-ox* and RSV *P2-ox* transgenic rice plants. Total protein was extracted from samples collected from 7-day-old seedlings and immunoblotted by gel blot with anti-SLR1 antibody. RbcL was used as a loading control. **(b)** QRT-PCR determined the transcription levels of *SLR1* in wild-type *Nip* rice seedlings and *SP8-ox*, *P2-ox* transgenic lines. **(c, d)** QRT-PCR determined the transcription levels of *SP8* **(c)** and *P2* **(d)** in wild-type *Nip* rice seedlings and *SP8-ox* or *P2-ox* transgenic lines.

**
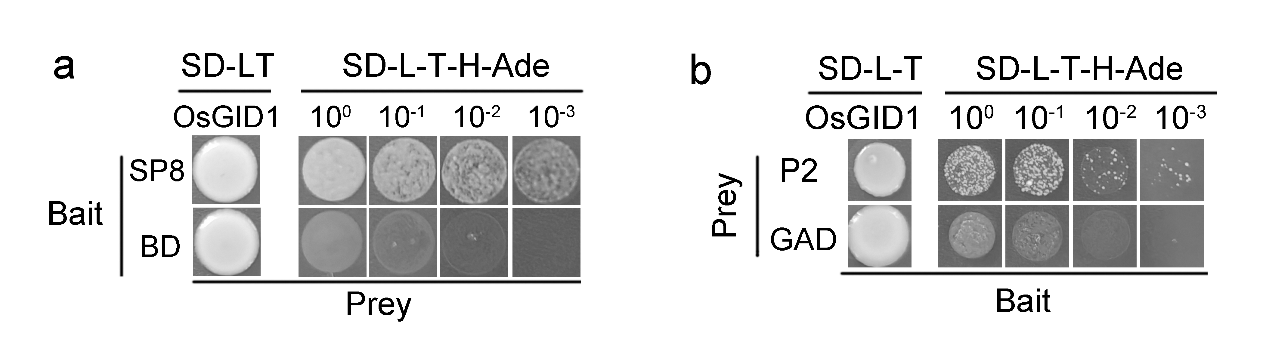
**

**Supplementary Figure 4. Viral proteins SP8 and P2 directly interact with OsGID1 receptor.** Y2H assays illustrating the direct interaction between OsGID1 with viral protein SP8 **(a)** and P2 **(b)**. Different combinations of constructs transformed into yeast cells were grown on SD-L-T-H-Ade plates at 30°C. Photos were taken after 3 days.

**
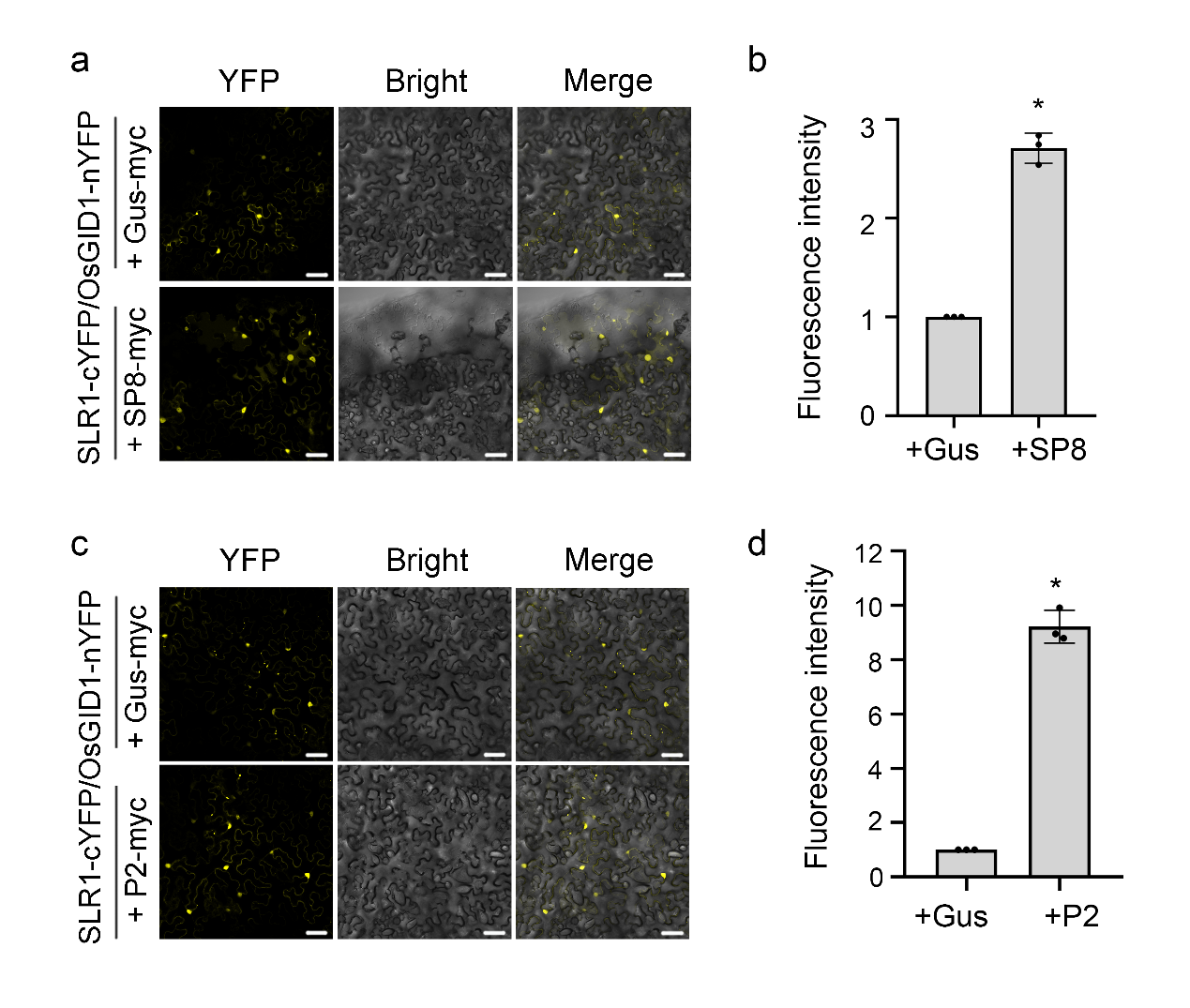
Supplementary Figure 5. Viral protein SP8 and P2 increase the association between OsSLR1 and OsGID1. (a, c)** Fluorescence from SLR1-cYFP and OsGID1-nYFP accumulated in *N. benthamiana* cells co-expressing SP8-myc **(a)** or P2-myc **(c)**. Gus-myc served as negative control. The images were observed by confocal microscopy at 48 hpi. Scale bar = 50 µm. **(b, d)** Quantification of the fluorescence intensity when SLR1-cYFP and OsGID1-nYFP were co-expressed with SP8-myc **(b)** or P2-myc **(d)**. Fluorescence intensity was analyzed by ImageJ. Data are means from at least 15 transfected *N. benthamiana* plants. Error bars represent SD and * indicates a significant difference between samples analyzed by ANOVA at p ≤ 0.05 using Fisher's least significant difference tests.

**
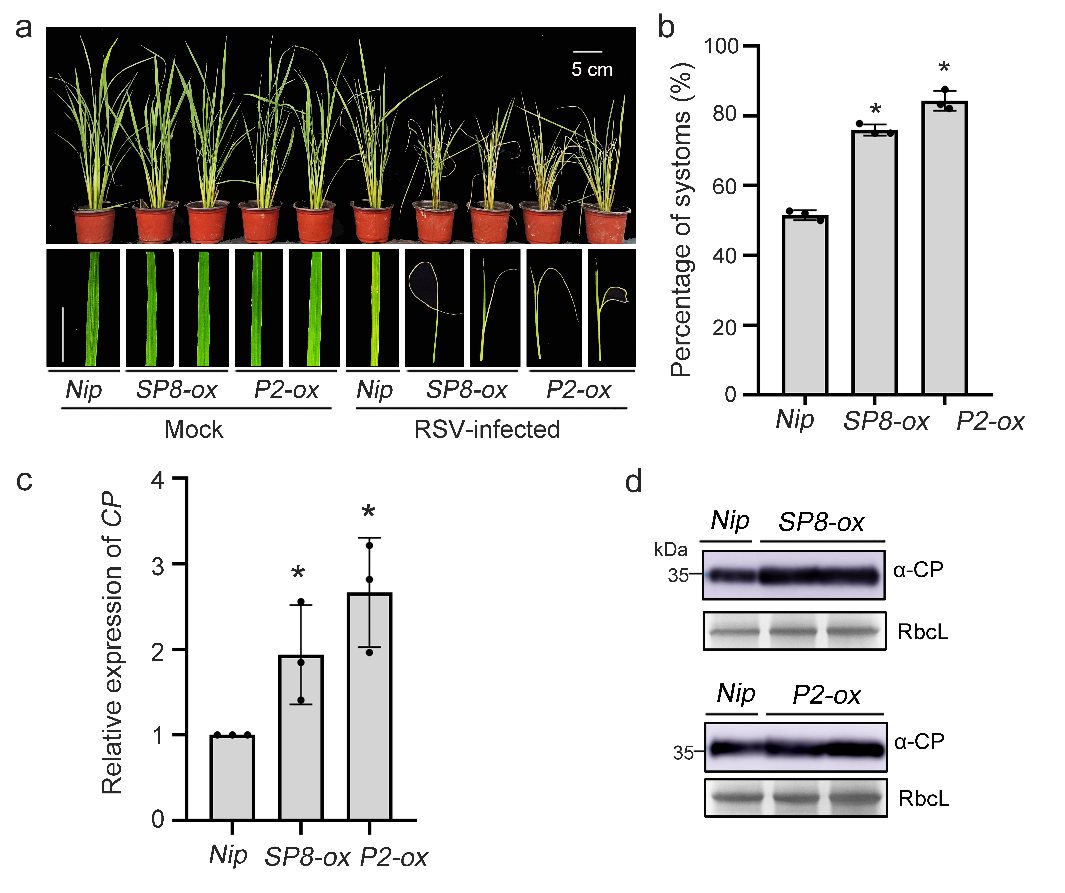
**

**Supplementary Figure 6. SP8 and RSV P2 negatively regulate rice antiviral defense against RSV. (a)** Plants and leaves of *Nip*, *SP8-ox* and *P2-ox* 20 d after inoculation with RSV. The areas of typical yellow stripes and curl or death of the young leaves represent the degree of disease symptoms. Phenotypes were obtained from at least three biological repeats, with 30 plants from each line in every repeat. Scale bars = 5 cm (upper panel) and 2 cm (lower panel). **(b)** Disease incidence and grades of symptoms in *Nip*, *SP8-ox* and *P2-ox* after inoculation with RSV. **(c)** Results of qRT-PCR showing the relative mRNA levels of RSV *CP* in RSV-infected *Nip*, *SSP8-ox* and *P2-ox* rice plants. * at the top of columns indicate significant differences (p < 0.05) based on student’s *t* test. **(d)** Western blot showing the accumulation of RSV CP protein in RSV-infected *Nip*, *SSP8-ox* and *P2-ox* rice plants. RbcL serves as the loading control.

**
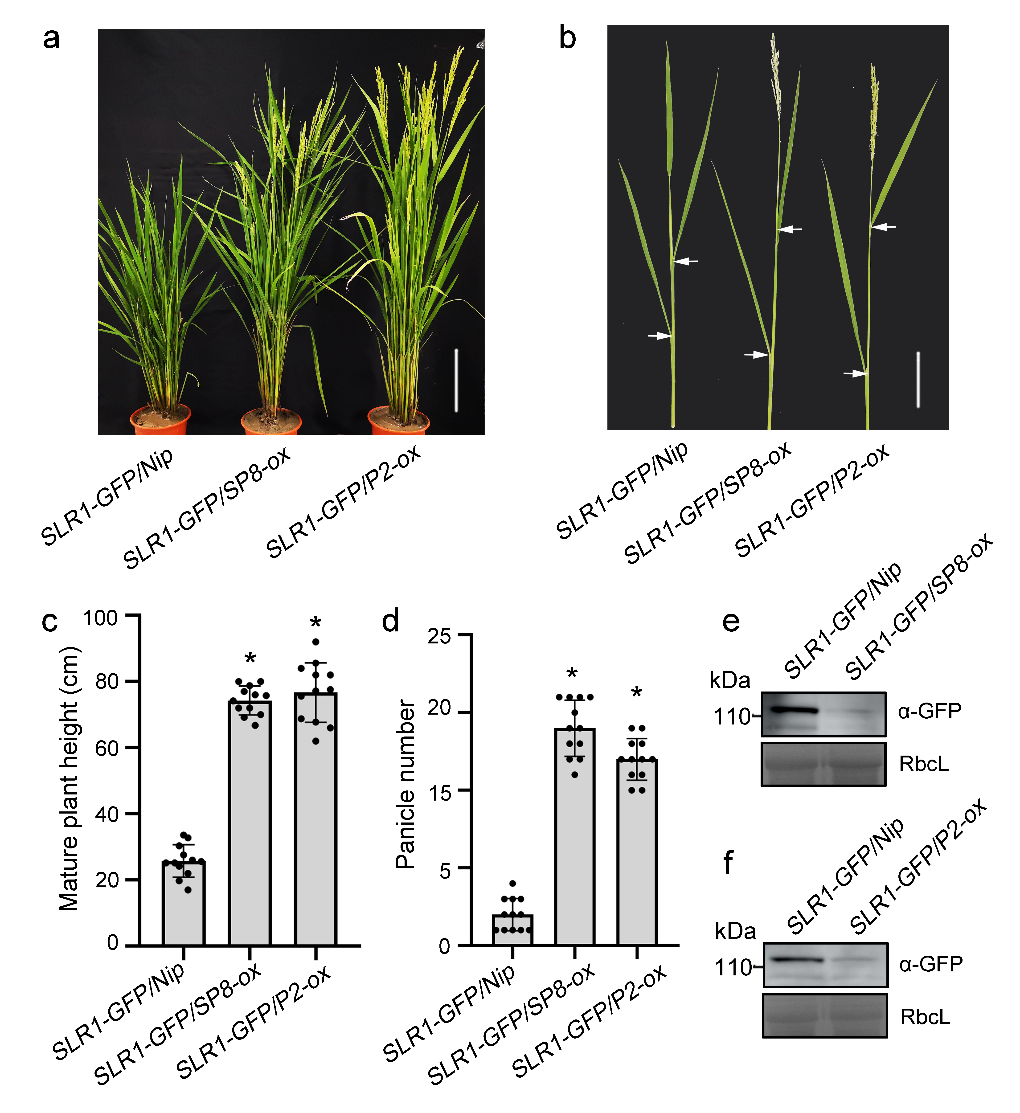
**

**Supplementary Figure 7.** **Phenotypes of different transgenic rice plants *SLR1-GFP*/*Nip*, *SLR1-GFP*/*SP8-ox* and *SLR1-GFP*/*P2-ox*.** **(a)** Images of *SLR1-GFP*/*Nip*, *SLR1-GFP*/*SP8-ox* and *SLR1-GFP*/*P2-ox* showing plant heights. The differences among the control and different transgenic plants is significant (p < 0.05) based on student’s *t* test. Scale bar = 8 cm. **(b)** Lengths of the second leaf sheath of *SLR1-GFP*/*Nip*, *SLR1-GFP*/*SP8-ox* and *SLR1-GFP*/*P2-ox* plants (from more than 10 plants of each line). Scale bar = 5 cm. **(c)** Mature plant height of *SLR1-GFP/Nip*, *SLR1-GFP/SP8-ox* and *SLR1-GFP/P2-ox*. Error bars represent SD, * indicates a significant difference between samples analyzed by ANOVA at *p* ≤ 0.05 by Fisher's least significant difference tests. **(d)** Panicle numbers in the transgenic rice plants *SLR1-GFP/Nip*, *SLR1-GFP/SP8-ox* and *SLR1-GFP/P2-ox*. Values were obtained from at least 10 seedlings. * at the top of columns indicate significant differences (p < 0.05) based on student’s t test. **(e, f)** Level of SLR1-GFP in different transgenic rice plants *SLR1-GFP/Nip*, *SLR1-GFP/SP8-ox* **(e)** and *SLR1-GFP/P2-ox* **(f)** using anti-GFP antibody. The samples were collected from 7-day-old seedlings for protein extraction, and RbcL was used as a loading control.

**
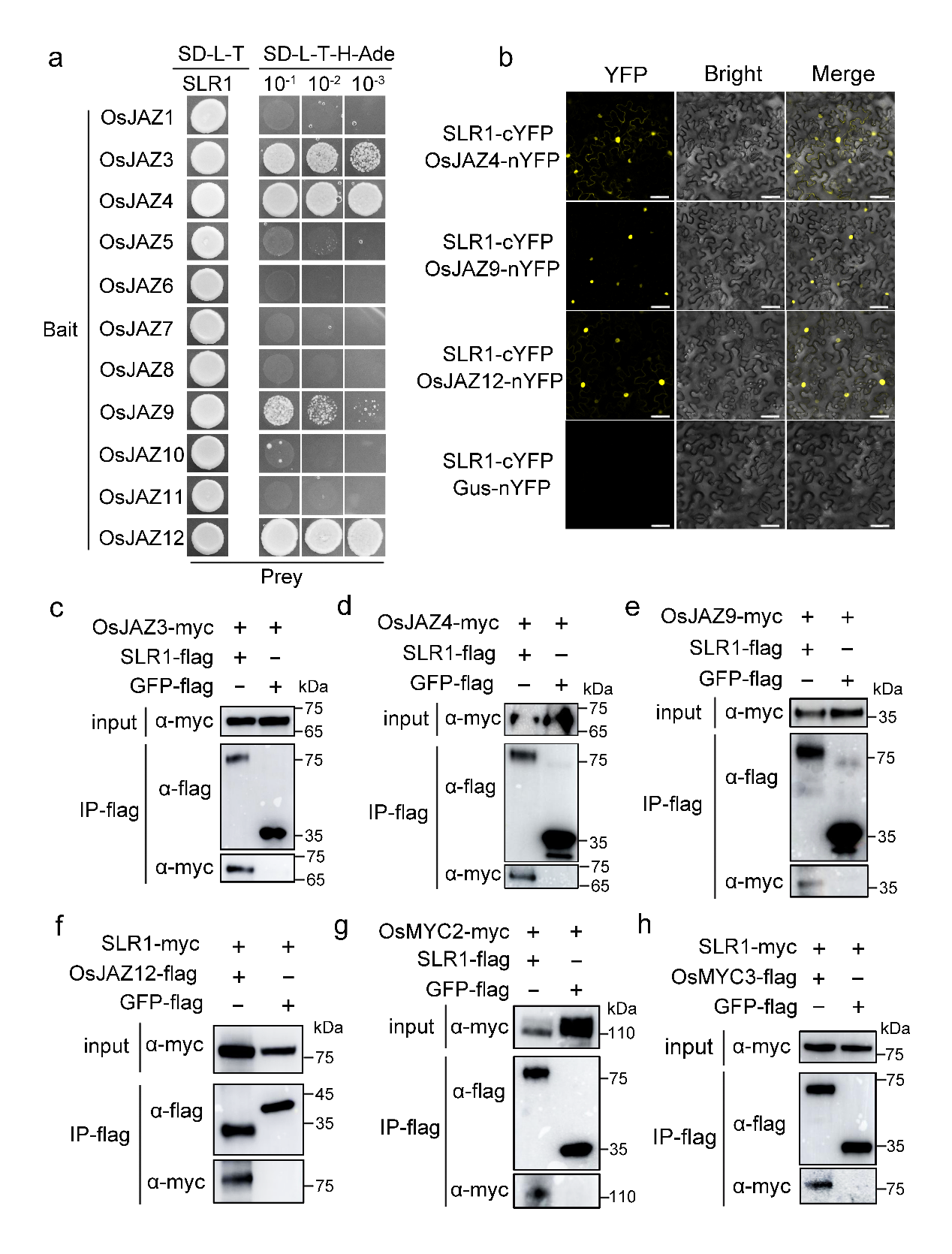
**

**Supplementary Figure 8. Crosstalk between SLR1 and JA pathway in host-virus interactions. (a)** Y2H assays showing the interaction between SLR1 and host OsJAZ proteins. Indicated plasmids were co-transformed into yeast strain AH109 and spotted on selected medium SD-L-T and SD-L-T-H-Ade at 30 °C. **(b)** BiFC images showing the interaction of SLR1 with OsJAZ proteins. Fluorescence appeared by complementation of the SLR1-cYFP with various OsJAZ-nYFPs. Gus-nYFP was used as a negative control. The samples were imaged by confocal microscopy at 48 hpi. Scale bar = 50 µm. **(c to h)** Co-IP experiments examining the interaction of SLR1 with various candidate proteins in *N. benthamiana* leaves, GFP-flag was used as a negative control. The samples were collected at 48 hpi, and then immunoprecipited with Flag- paramagnetic beads.

**
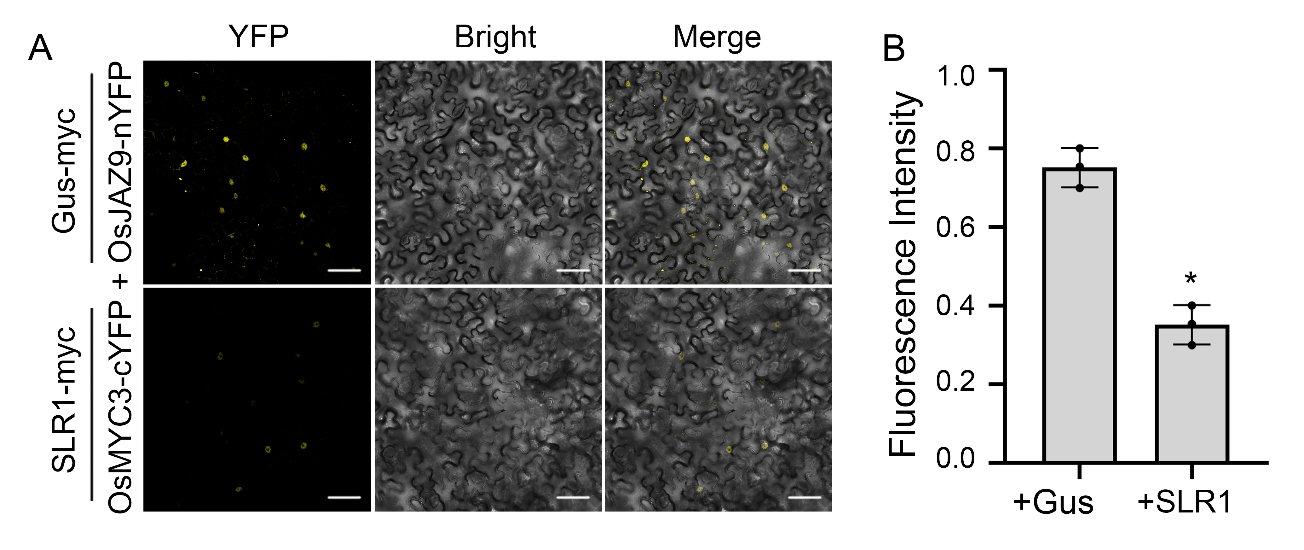
Supplementary Figure 9.** **SLR1 disturbs the association between OsJAZ9 and OsMYC3. (a)** Fluorescence from OsMYC3-cYFP and OsJAZ9-nYFP accumulated in *N. benthamiana* cells co-expressing Gus-myc or SLR1-myc. The images were observed by confocal microscopy at 48 hpi. Scale bar = 50 µm. **(b)** Quantification of the fluorescence intensity when OsMYC3-cYFP and OsJAZ9-nYFP were co-expressed with Gus-myc or SLR1-myc. Fluorescence intensity was analyzed by ImageJ. Data are means from at least 15 transfected *N. benthamiana* plants. Error bars represent SD and * indicates a significant difference between samples analyzed by ANOVA at *p* ≤ 0.05 using Fisher's least significant difference tests.


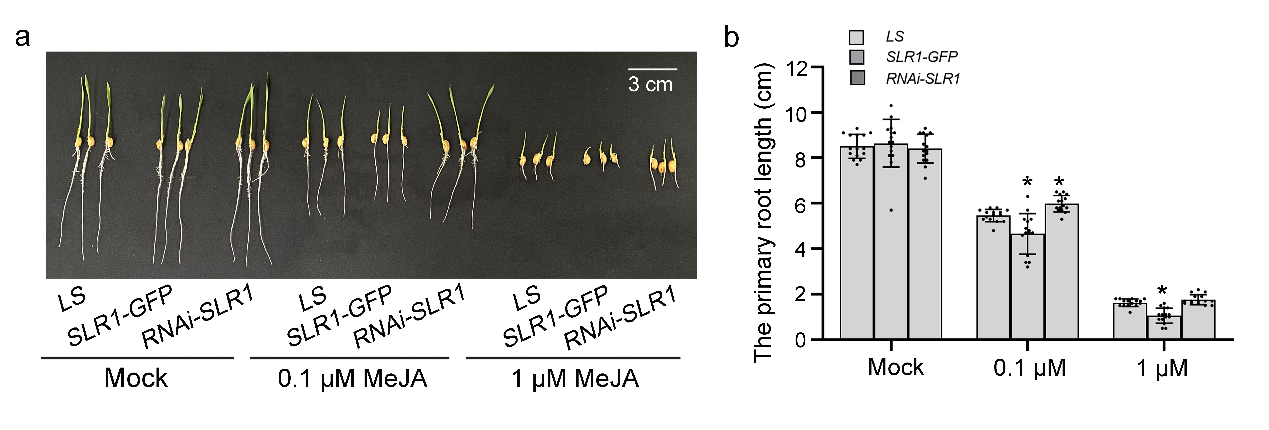


**Supplementary Figure 10. JA sensitivity of plants overexpressing SLR1 and *RNAi-SLR1* mutants. (a)** Phenotypes of *LS*, *SLR-GFP* and *RNAi-SLR1* seedlings treated with MeJA. Similar germinated seeds were planted in different concentrations of MeJA (0, 0.1, 1 µM) containing culture solution for about 7 d. Images were photographed using a digital camera. Scale bar = 3 cm. **(b)** Root lengths of *LS*, *SLR1-GFP* and *RNAi-SLR1* seedlings. Error bars represent SD and * indicates a significant difference between samples analyzed by ANOVA at *p* ≤ 0.05 using Fisher's least significant difference tests. Statistical analysis was based on at least three biological repeats, with 15 plants from each line in every repeat.

**
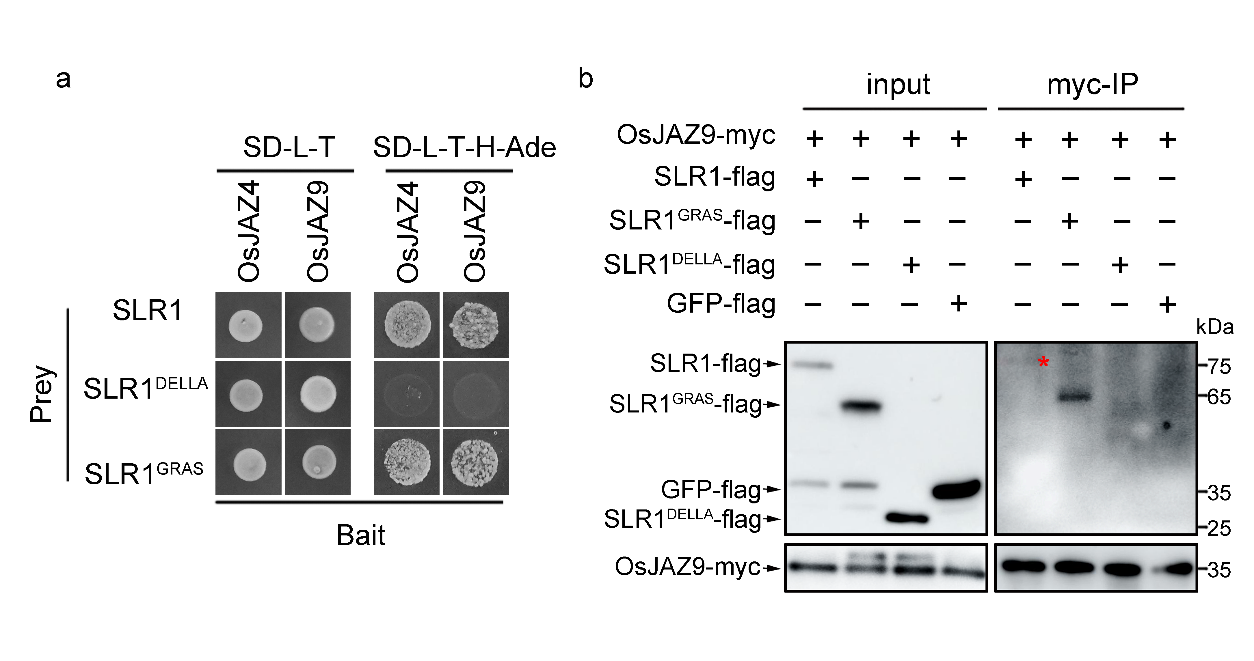
**

**Supplementary Figure 11. Mapping the domain of SLR1 that interacts with OsJAZ proteins. (a)** The conserved GRAS domain of SLR1 is required for interaction with OsJAZ4 and OsJAZ9 proteins. Schematic diagrams of DELLA or GRAS mutants of SLR1 are shown in Fig 1a. Different derivatives and host OsJAZ proteins were respectively fused with the AD and BD yeast vectors. Positive transformants were selected on SD-L-T-H-Ade plates and photographed after 3 days. **(b)** Co-IP assay showing that SLR1 interact with OsJAZ9 through GRAS domain *in vivo*. Total proteins were extracted from N. benthamiana leaves expressing OsJAZ9-myc with different SLR1 mutants, the supernatant was precipitated with myc beads and the associated proteins were verified using anti-flag antibody. The sample co-expressing OsJA9-myc with SLR1-flag acts as a positive control while GFP-flag serves as a negative control.

**
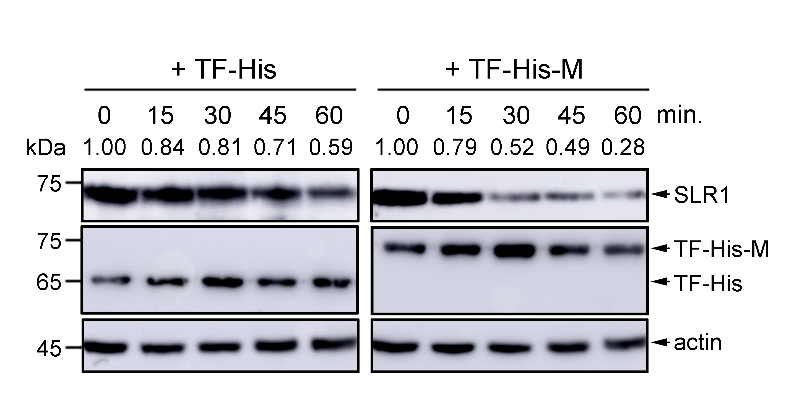
**

**Supplementary Figure 12. RSMV M protein promotes the degradation of SLR1 in cell-free system.** Time course of SLR1 degradation in the wild-type *Nip* protein extracts treated with TF-His or TF-His-M. Equal amounts of plant crude extracts were added to equal amounts of the recombinant proteins in the in vitro cell-free degradation assays. The plant actin antibody was used as a loading control.

**
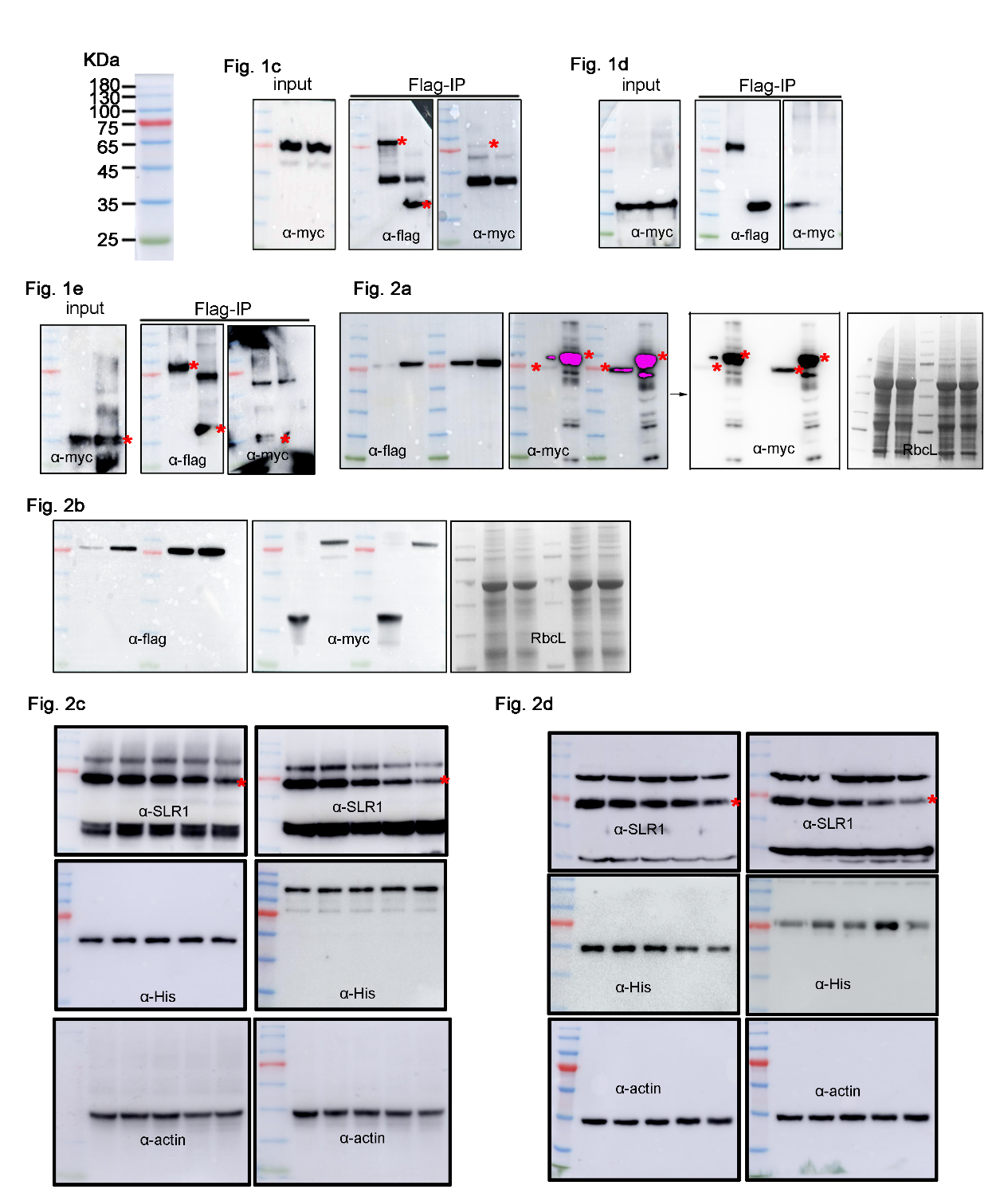
**

**Supplementary Figure 13. Original images for immunoblots shown in Fig.1 to Fig.2.**

**
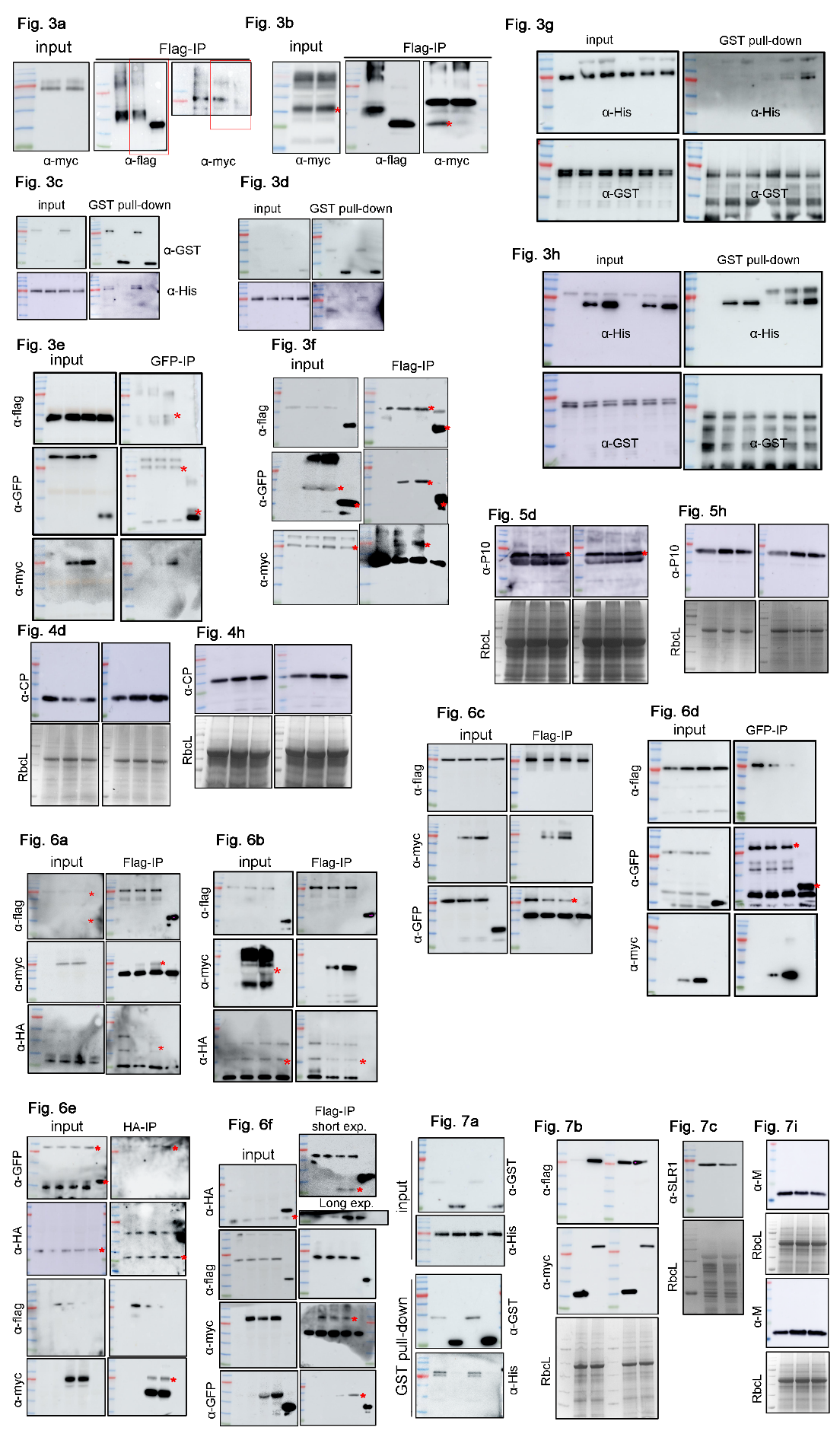
Supplementary Figure 14. Original images for immunoblots shown in Fig.3 to Fig.7.**

**
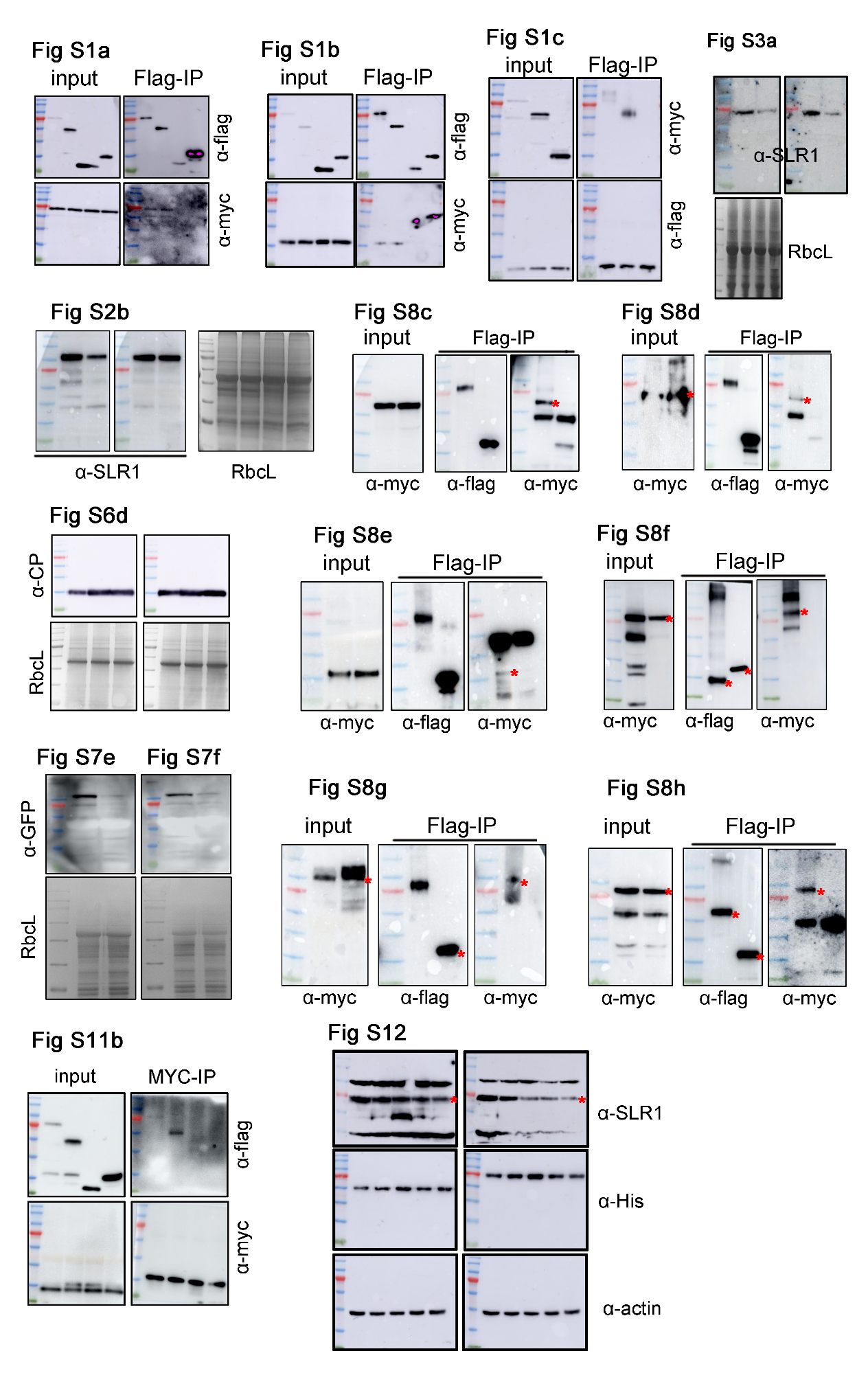
**

**Supplementary Figure 15. Original images for immunoblots shown in Supplementary Fig.1-12.**

**Supplementary Table 1.** **The primers used in this study.**

| **Gene Name** | **Primer Name** | **Sequence 5’-3’** |
| --- | --- | --- |
| **For Yeast Two-Hybrid (Y2H) Assays** | | |
| SLR1 | AD-SLR1-F | GACGTACCAGATTACGCTCATATGAAGCGCGAGTACCAAGAA |
|  | AD-SLR1-R | GCAGCTCGAGCTCGATGGATCCttaCGCCGCGGCGACGCGCCA |
| SLR1 truncated mutants | AD-SLR1^DELLA^-F | GACGTACCAGATTACGCTCATATGAAGCGCGAGTACCAAGAA |
|  | AD-SLR1^DELLA^-R | GCAGCTCGAGCTCGATGGATCCttaAGCCGACGGGTCAGCCGT |
|  | AD-SLR1^GRAS^-F | GACGTACCAGATTACGCTCATATGGCTGACTCGGCGAGG |
|  | AD-SLR1^GRAS^-R | GCAGCTCGAGCTCGATGGATCCttaCGCCGCGGCGACGCGCCA |
| OsGID1 | AD-OsGID1-F | GACGTACCAGATTACGCTCATATGGCCGGCAGCGACGA |
|  | AD-OsGID1-R | GCAGCTCGAGCTCGATGGATCCttaGTAGTAGAGGTTAGCGTT |
| SRBSDV | BD-SP8-F | ATCTCAGAGGAGGACCTGCATATGATGATCGGTACATACGATGAT |
|  | BD-SP8-R | GCCGCTGCAGGTCGACGGATCCTTAACACAGAATACTAACGGCG |
| RBSDV | BD-RBP8-F | ATCTCAGAGGAGGACCTGCATATGATGACTGGCACCCATGACGA |
|  | BD-RBP8-R | GCCGCTGCAGGTCGACGGATCCTTATACAATAATCGAGGAAGCT |
| RSV | BD-P2-F | ATCTCAGAGGAGGACCTGCATATGATGGCATTACTCCTCTTCAAT |
|  | BD-P2-R | GCCGCTGCAGGTCGACGGATCCTTACATTAGAATAGGGCACT |
| RSMV | BD-M-F | ATCTCAGAGGAGGACCTGCATATGATGGCCGTTCCGTGGACT |
|  | BD-M-R | GCCGCTGCAGGTCGACGGATCCCTAACTCCAGATTATACTTCC |
| OsJAZ homologues | BD-OsJAZ1-F | ATCTCAGAGGAGGACCTGCATATGGATCTGTTGGAGAAGAAG |
|  | BD-OsJAZ1-R | GCCGCTGCAGGTCGACGGATCCTTACTGGGCCTTGCCCTCAG |
|  | BD-OsJAZ3-F | ATCTCAGAGGAGGACCTGCATATGGAGAGGGATTTTCTTGG |
|  | BD-OsJAZ3-R | GCCGCTGCAGGTCGACGGATCCTCATATCTGTAACTTTGTGCTG |
|  | BD-OsJAZ4-F | ATCTCAGAGGAGGACCTGCATATGGAGAGGGACTTCCTGG |
|  | BD-OsJAZ4-R | GCCGCTGCAGGTCGACGGATCCCTAGATTTGTAGCTTTGTACTG |
|  | BD-OsJAZ5-F | ATCTCAGAGGAGGACCTGCATATGTCGACGAGGGCGCC |
|  | BD-OsJAZ5-R | GCCGCTGCAGGTCGACGGATCCCTAGGACGCCGTGTGCTC |
|  | BD-OsJAZ6-F | ATCTCAGAGGAGGACCTGCATATGGCTTCCGCGAAATCCG |
|  | BD-OsJAZ6-R | GCCGCTGCAGGTCGACGGATCCTCATTGGCTCGATTCCTGC |
|  | BD-OsJAZ7-F | ATCTCAGAGGAGGACCTGCATATGGCGGCTTCCGCGAG |
|  | BD-OsJAZ7-R | GCCGCTGCAGGTCGACGGATCCTCATTGGCCGCGTTCTATG |
|  | BD-OsJAZ8-F | ATCTCAGAGGAGGACCTGCATATGGCCGGCCGTGCGAC |
|  | BD-OsJAZ8-R | GCCGCTGCAGGTCGACGGATCCTCATATCTCCTGCTTTATT |
|  | BD-OsJAZ9-F | ATCTCAGAGGAGGACCTGCATATGGCGTCGACGGATCCC |
|  | BD-OsJAZ9-R | GCCGCTGCAGGTCGACGGATCCTCAGCGCGAGTGCATGTGT |
|  | BD-OsJAZ10-F | ATCTCAGAGGAGGACCTGCATATGGCGATGGAGGGGAAGA |
|  | BD-OsJAZ10-R | GCCGCTGCAGGTCGACGGATCCTCACAGCGCGATGGTGAG |
|  | BD-OsJAZ11-F | ATCTCAGAGGAGGACCTGCATATGGCCGGTAGTAGCGAG |
|  | BD-OsJAZ11-R | GCCGCTGCAGGTCGACGGATCCTCACAGGCTGAGAGTGGG |
|  | BD-OsJAZ12-F | ATCTCAGAGGAGGACCTGCATATGGCCGCCGCCGGCA |
|  | BD-OsJAZ12-R | GCCGCTGCAGGTCGACGGATCCCTAGAGCCCGAGCCATGT |
| OsGID1 | BD-OsGID1-F | ATCTCAGAGGAGGACCTGCATATGGCCGGCAGCGACGA |
|  | BD-OsGID1-R | GCCGCTGCAGGTCGACGGATCCCTAGTAGTAGAGGTTAGCGTT |
| **For construction of overexpression transgenic rice plants** | | |
| SP8-ox | Lic-SP8-F | CgACgACAAgACCgTCACCATGATCGGTACATACGATGAT |
|  | Lic-SP8-R | gAggAgAagAgCCgTCgACACAGAATACTAACGGCG |
| P2-ox | Lic-P2-F | CgACgACAAgACCgTCACCATGGCATTACTCCTCTTCAAT |
|  | Lic-P2-R | gAggAgAagAgCCgTCgCATTAGAATAGGGCACT |
| **For qRT-PCR** | | |
| SLR1-ox^1^ | qRT-SLR1-F | GCTCCAATGCCTACAAACA |
|  | qRT-SLR1-R | TTCTCCTCCACCCGGTAG |
| SP8-ox^2^ | qRT-SP8-F | CGTTCGTACCTCATTCGCTG |
|  | qRT-SP8-R | GAAAAGCGGGAGTGGGATTC |
| P2-ox^2^ | qRT-P2-F | GGTCAACGGGTTTCAGTTTGC |
|  | qRT-P2-R | GGTCAGGGAGAGCAATTTCG |
| SRBSDV^2, 3^ | qRT-S2-F | CATCGACCAAGTTCAACCCG |
|  | qRTS2-R | AAGAAGTCTGCGGGTGAAGA |
|  | qRT-S4-F | AAAGTGAACCCGTTGCTGAC |
|  | qRT-S4-R | TGCAACGCTAGATCCTATGC |
|  | qRT-S6-F | ATCTGCTTTTCCCCTTCCGA |
|  | qRT-S6-R | GATTCCGCGTTTGAAGAGTCA |
| RSMV^3^ | qRT-N-F | AGAGGTTGGAGAGGGGAAGA |
|  | qRT-N-R | TAGCCGCCCTTCTATCCTTG |
|  | qRT-M-F | ACTTCAGTGTCCAGCCTACC |
|  | qRT-M-R | CCTCCATCCACCTTGCACTC |
| RSV^2, 3^ | qRT-CP-F | AGGCAATCAATGACATCTCC |
|  | qRT-CP-R | ATCTCTCACAAAGCCAGTGC |
| **For BiFC and Co-IP Assays** | | |
| SLR1 | Lic-SLR1-F | CgACgACAAgACCgTCACCATGAAGCGCGAGTACCAAGAA |
|  | Lic-SLR1-R | gAggAgAagAgCCgTCgCGCCGCGGCGACGCGCCA |
| SRBSDV P8 | Lic-SP8-F | CgACgACAAgACCgTCACCATGATCGGTACATACGATGAT |
|  | Lic-SP8-R | gAggAgAagAgCCgTCgACACAGAATACTAACGGCG |
| RSV P2 protein | Lic-P2-F | CgACgACAAgACCgTCACCATGGCATTACTCCTCTTCAAT |
|  | Lic-P2-R | gAggAgAagAgCCgTCgCATTAGAATAGGGCACTCA |
| RSMV M protein | Lic-M-F | CgACgACAAgACCgTCACCATGGCCGTTCCGTGGACT |
|  | Lic-M-R | gAggAgAagAgCCgTCgACTCCAGATTATACTTCCTCC |
| OsGID1 | Lic-OsGID1-F | CgACgACAAgACCgTCACCATGGCCGGCAGCGACGA |
|  | Lic-OsGID1-R | gAggAgAagAgCCgTCgGTAGTAGAGGTTAGCGTT |
| OsJAZ homologues | Lic-OsJAZ3-F | CgACgACAAgACCgTCACCATGGAGAGGGATTTTCTTGG |
|  | Lic-OsJAZ3-R | gAggAgAagAgCCgTCgTATCTGTAACTTTGTGCTG |
|  | Lic-OsJAZ4-F | CgACgACAAgACCgTCACCATGGAGAGGGACTTCCTGG |
|  | Lic-OsJAZ4-R | gAggAgAagAgCCgTCgGATTTGTAGCTTTGTACTG |
|  | Lic-OsJAZ9-F | CgACgACAAgACCgTCACCATGGCGTCGACGGATCCC |
|  | Lic-OsJAZ9-R | gAggAgAagAgCCgTCgGCGCGAGTGCATGTGT |
|  | Lic-OsJAZ12-F | CgACgACAAgACCgTCACCATGGCCGCCGCCGGCA |
|  | Lic-OsJAZ12-R | gAggAgAagAgCCgTCgGAGCCCGAGCCATGT |
| OsMYC homologues | Lic-OsMYC2-F | CgACgACAAgACCgTCACCATGTGGGTTTTGTTATCTCCT |
|  | Lic-OsMYC2-R | gAggAgAagAgCCgTCgCCGGGCGGCGGTGCC |
|  | Lic-OsMYC3-F | CgACgACAAgACCgTCACCATGTCGTGGTCCGAGACG |
|  | Lic-OsMYC3-R | gAggAgAagAgCCgTCgTGGAGATGGTGTAGTAAC |
| **For protein purification** | | |
| SRBSDV P8 | His-SP8-F | gtggtatcgaaggtaggcatatgATCGGTACATACGATGAT |
|  | His-SP8-R | ACAAGCTTGAATTCGGATCCttaACACAGAATACTAACG |
|  | GST-SP8-F | TCCAGGGGCCCCTGGGATCCATGATCGGTACATACGATGAT |
|  | GST-SP8-R | GTCAGTCACGATGCGGCCGCttaACACAGAATACTAACG |
| RSV P2 | His-P2-F | gtggtatcgaaggtaggcatatgATGGCATTACTCCTCTTCAAT |
|  | His-P2-R | ACAAGCTTGAATTCGGATCCttaCATTAGAATAGGGCACTCA |
|  | GST-P2-F | TCCAGGGGCCCCTGGGATCCATGGCATTACTCCTCTTCAAT |
|  | GST-P2-R | GTCAGTCACGATGCGGCCGCttaCATTAGAATAGGGCACTCA |
| RSMV-M | His-M-F | gtggtatcgaaggtaggcatatgATGGCCGTTCCGTGGACT |
|  | His-M-R | ACAAGCTTGAATTCGGATCCttaACTCCAGATTATACTTCCTCC |
|  | GST-M-F | TCCAGGGGCCCCTGGGATCCATGGCCGTTCCGTGGACT |
|  | GST-M-R | GTCAGTCACGATGCGGCCGCttaACTCCAGATTATACTTCCTCC |
| SLR1 | GST-SLR-F | TCCAGGGGCCCCTGGGATCCATGAAGCGCGAGTACCAAGAA |
|  | GST-SLR1-R | GTCAGTCACGATGCGGCCGCttaCGCCGCGGCGACGCGCCA |
| OsGID1 | His-OsGID1-F | gtggtatcgaaggtaggcatatgATGGCCGGCAGCGACGA |
|  | His-OsGID1-R | ACAAGCTTGAATTCGGATCCttaGTAGTAGAGGTTAGCGTT |

**Reference**

1. Liao Z*, et al.* SLR1 inhibits MOC1 degradation to coordinate tiller number and plant height in rice. *Nature Communications* **10**, 2738 (2019).

2. Li L*, et al.* A class of independently evolved transcriptional repressors in plant RNA viruses facilitates viral infection and vector feeding. *Proceedings of the National Academy of Sciences* **118**, e2016673118 (2021).

3. Zhang H*, et al.* Distinct modes of manipulation of rice auxin response factor OsARF17 by different plant RNA viruses for infection. *Proceedings of the National Academy of Sciences* **117**, 9112-9121 (2020).
